# Associations of methylmalonic acid and depressive symptoms with mortality: a population-based study

**DOI:** 10.1038/s41398-024-03015-6

**Published:** 2024-07-19

**Authors:** Bing Cao, Yefei Xiao, Dan Liu

**Affiliations:** 1 Key Laboratory of Cognition and Personality, Faculty of Psychology, Ministry of Education, Southwest University, Chongqing, 400715 P. R. China; 2 National Demonstration Center for Experimental Psychology Education, Southwest University, Chongqing, 400715 P. R. China; 3 Population Health Sciences, German Center for Neurodegenerative Diseases (DZNE), Bonn, Germany

**Keywords:** Depression, Biomarkers

## Abstract

Methylmalonic acid (MMA), a biomarker of mitochondrial dysfunction, has been reported to be associated with depression in specific populations (i.e., older adults and postpartum women). Our study aimed to investigate to what extent MMA was associated with depressive symptoms and mortality in the general population, and assess whether depressive symptoms mediate the relationship between MMA and mortality. We analyzed cross-sectional data from 8343 participants from the US National Health and Nutrition Examination Survey. MMA was measured by liquid chromatography-tandem mass spectrometry, while depressive symptoms were measured by the Patient Health Questionnaire-9. Mortality data were obtained through linkage with National Death Index records. Linear regression models were performed to assess the association between MMA and depressive symptoms. The Cox proportional hazard regression model was utilized to assess the association of MMA and depressive symptoms with mortality. Mediation analysis was conducted within the counterfactual framework. In this general population, each SD (around 0.49 μmol/L) increase in MMA was associated with a 0.03 SD (approximately 0.15 score) increase in depressive symptoms (β = 0.033, 95% CI: 0.010, 0.055, *p* = 0.005). Notably, this association was more pronounced in men and participants over 60 years old. Higher levels of MMA and having more depressive symptoms were associated with a higher risk of mortality. However, depressive symptoms do not mediate the relationship between MMA and mortality. Elevated MMA levels were associated with depressive symptoms and an increased risk of mortality. These findings suggest that mitochondrial dysfunction may contribute to the multifactorial etiology of depression.

## Introduction

Depression is one of the most common mental disorders [1], and has been related to all-cause and cardiovascular disease mortality among adults [2, 3]. Thus, it becomes essential to elucidate the etiopathogenesis of depression. It has been acknowledged that neurobiological, psychological, social, and genetic factors contribute to the pathogenesis of depression [4]. Recent evidence shows that mitochondrial dysfunction [5] and oxidative stress [6] contribute to the pathogenesis of depression.

Methylmalonic acid (MMA) is a mitochondrial toxin that may hamper mitochondrial energy metabolism by interfering with succinate dehydrogenase and triggering reactive oxygen species generation [7, 8]. Indeed, recent animal studies have shown that MMA can disrupt oxidative stress and energy metabolism in the brain [9–11]. Epidemiological studies have found that MMA was associated with depression in older and/or postpartum women [12, 13], cognition impairment [14–16], and mortality [17]. Moreover, MMA is a biomarker of vitamin B12 deficiency since vitamin B12 deficiency may prevent the conversion of MMA to succinyl CoA [18]. Due to the role of vitamins B12 and folate in one-carbon metabolism, their deficiency may affect MMA methylation in the central nervous system, leading to depression [19–22]. However, the exact relationship between MMA and depressive symptoms in the general population across the adult lifespan remains unclear. In addition, whether and to what extent this association is independent of vitamin B12 and folate is largely unknown.

Therefore, this study aimed to investigate associations between MMA and depressive symptoms in adults across a wide age spectrum and whether this association was independent of vitamin B12 and folate. This study also examined whether the association between MMA and depressive symptoms would differ by age and sex. Additionally, the association of MMA and depressive symptoms with mortality and the mediating role of depressive symptoms in the relationship between MMA and mortality was assessed.

## Methods

### Study sample

The National Health and Nutrition Examination Survey (NHANES) is a cross-sectional study that includes a nationally representative sample of the non-institutionalized, civilian population in the US. It is an ongoing program with a 2-year reporting cycle that is managed by the National Center for Health Statistics (NCHS) of the Centers for Disease Control and Prevention (CDC). Data collection within NHANES is methodically structured as a sequential two-stage procedure. Initially, participants engage in a comprehensive in-home interview to record demographic details, socioeconomic status, dietary habits, and health history. This is followed by a detailed examination at a Mobile Examination Center (MEC), where a suite of medical and laboratory tests administered, along with physical measurements to gather clinical data. Our study utilized the comprehensive dataset that included results from clinical and laboratory tests, dietary assessments, and detailed health-related questionnaires. The NCHS Research Ethics Review Board endorsed the study protocol, and all participants provided written informed consent. More information is available on the NHANES website (cdc.gov/nchs/nhanes.htm).

For this analysis, data from the study cycles 2011–2012 and 2013–2014 were combined, resulting in an initial sample size of 19,931 individuals. Subsequently, the analysis was focused on 11,977 adult participants aged 18 and above. Of these, 10,913 provided completed data on depressive symptoms. We further excluded participants with missing data on MMA (*n* = 1008), mortality (*n* = 12) or covariates (i.e., age, sex, race/ethnicity, education, poverty income ratio, marital status, body max index, vitamin B12, folate, mean cell volume (MCV) and hematocrit) (*n* = 974). Consequently, the final sample for the present study comprised 8343 individuals. Figure S1 displays a thorough flowchart of the participant selection process.

### Measurement of MMA

MMA was determined in the serum using liquid chromatography-tandem mass spectrometry (LC-MS/MS) as dibutylester. Blood samples were collected using venipuncture in the MECs, following the established standards of the NHANES. MMA was extracted from a 75 μL sample of serum together with an internal standard (d3-MMA). After butanol derivatization, MMA was measured by LC-MS/MS with multiple reaction monitoring and quantitated by the peak area ratios of MMA and isotope-labeled d3-MMA, using the units of μmol/L.

### Measurement of depressive symptoms

Depressive symptoms were assessed using the Patient Health Questionnaire-9 (PHQ-9), which evaluated the frequency of specific symptoms experienced by individuals within the past two weeks. The computer-assisted personal interview was administered to all NHANES participants aged 12 and above. The PHQ-9 comprises a set of nine items that individuals respond to using a four-point Likert scale, from 0 (“indicating no occurrence”) to 3 (“suggesting near-daily occurrence”) [23]. The total score was calculated by summing the responses to all PHQ questions, ranging from 0 to 27.

### Measurement of mortality

The all-cause mortality status of the participants was ascertained through a probabilistic matching process between the NHANES dataset and the National Death Index (NDI) death certificate records. The survival time (months) is determined as the period starting from the NHANES examination date until the occurrence of death. For participants who survived until the end of the follow-up period (December 31, 2019), the survival time was calculated by subtracting the date of survey participation from this endpoint.

### Measurement of covariates

Covariates included sociodemographic variables, physical information, and biomedical data. All sociodemographic variables were collected from the questionnaire investigation. Race was coded as Mexican American, Other Hispanic, Non-Hispanic White, Non-Hispanic Black, and Other Race. Education was classified as less than high school education attainment, high school graduate (has a high school diploma or high school equivalency diploma such as a General Educational Development), and more than high school education. The poverty-to-income ratio was an index for the ratio of family income to poverty and was calculated by dividing family income by poverty guidelines specific to family size, year, and state. Marital status was defined as married, not married, and other status (separated, widowed, or divorced). Additional information is available in the NHANES Procedure Manual.

Both physical examinations and blood sampling were performed in MECs. Body mass index (BMI) was calculated as weight in kilograms divided by height in meters squared (kg/m2). Serum vitamin B-12 was measured by the Elecsys Vitamin B-12 assay, employing a competitive electrochemiluminescence immunoassay (“ECLIA”). Red blood cell (RBC) folate was used to measure long-term folate status, calculated utilizing the data from microbiologic assay and LC-MS/MS. Also, serum folate was measured by LC-MS/MS, as an indicator of more recent folate intake. In addition, dietary intakes of vitamin B-12 and folate were considered as covariates in the study. MCV is measured by the Beckman Coulter MAXM equipment. Hematocrit was determined using a quantitative, automated hematology analyzer. The details can be found in the NHANES Laboratory/Medical Technologists Procedures Manual.

### Statistical analysis

The basic characteristics of the participants were described using proportions and percentages (%) or means ± standard deviations (SD). The concentrations of MMA were subjected to a natural logarithm transformation before analysis. Independent samples t-test and chi-square test were employed for continuous and categorical variables, respectively. Pearson correlation coefficients were employed to quantify the degree of correlation among the principal variables. To enhance the comparability of the results, crucial variables including log MMA, PHQ-9 score, BMI, vitamin B12, folate, MCV, and hematocrit were standardized before further analysis.

Multivariable linear regression analyses were used to assess the association between MMA with depressive symptoms. Model 1 was adjusted for age (continuous), sex (male or female), race (Mexican American, other Hispanic, Non-Hispanic White, Non-Hispanic Black, or other race), education (less than high school, high school graduate, or more than high school), marital status (married, never married, or other status) and poverty to income ratio. BMI was adjusted in Model 2 and Model 3 incorporated the adjustment for vitamin B12 and folate. In Model 4, MCV and hematocrit were further adjusted. Each subsequent model is built upon the previous model by incorporating additional variables. Subgroup analyses were performed according to sex (men and women) and age (< 60, and ≥ 60 years). A sensitivity analysis was conducted to explore the associations between MMA, depression, and mortality, adjusted for dietary intakes of vitamin B12 and folate.

Moreover, multivariable Cox proportional hazard regression models were utilized to assess the hazard ratios (HRs) and 95% CIs for the association of MMA and depressive symptoms with all-cause mortality. The Cox regression models were adjusted for age (continuous), sex (male or female), race (Mexican American, other Hispanic, Non-Hispanic White, Non-Hispanic Black, or other race), education (less than high school, high school, or more than high school), marital status (married, never married, or other status), BMI, vitamin B12, folate, MCV, and hematocrit. To evaluate to what extent depressive symptoms account for the association between MMA and mortality, we adjusted for depressive symptoms in the Cox regression model of MMA with mortality. The proportion of risk explained by depressive symptoms was estimated using the following formula: [(HR _basic model_ – HR _adjusted_) / (HR _basic model_ − 1) × 100] %, where HR represents the hazard ratio [24, 25]. We further use the counterfactual approach developed by Valeri and VanderWeele [26, 27] to examine the mediating role of depressive symptoms in the relationship between MMA and mortality. This approach allows for direct and indirect causal effects from causal inference in the counterfactual framework. All statistical analyses were performed by using SAS version 9.4 (SAS Institute Inc., Cary, NC, USA) and R version 4.3.0 (R Core Team 2020) with a 2-sided *P* < 0 .05 considered statistically significant.

## Results

### Demographic characteristics of the study population

Table 1 presents the characteristics of the study subjects. Among 8343 individuals, the average age was 48.8 ± 17.6 years and 50.4% (*n* = 4207) of participants were women. The mean PHQ-9 score was 3.3 ± 4.6. Compared to men, women have higher scores of depressive symptoms, lower levels of MMA, higher levels of BMI, vitamin B12, folate, MCV, and hematocrit. Supplementary Fig. 2 shows the Pearson correlation matrix among MMA, PHQ-9, BMI, vitamin B12, folate, MCV, and hematocrit. However, no significant correlations were found among these variables (correlation coefficients ranging from -0.17 to 0.26).

**Table 1:** Characteristics of study sample (NHANES 2011-2014).

	Overall	Men	Women	*P* value
	*N* = 8343	*N* = 4136	*N* = 4207	
AGE, years	48.77 (17.59)	48.47 (17.66)	49.08 (17.53)	0.114
**RACE, n (%)**				0.102
Mexican American	921 (11.04%)	480 (11.61%)	441 (10.48%)	
Other Hispanic	776 (9.30%)	358 (8.66%)	418 (9.94%)	
Non-Hispanic White	3588 (43.00%)	1790 (43.28%)	1798 (42.74%)	
Non-Hispanic Black	1852 (22.20%)	897 (21.69%)	955 (22.70%)	
Other Race	1206 (14.46%)	611 (14.77%)	595 (14.14%)	
**Education, n (%)**				<0.001
Less than high school education attainment	1688 (20.23%)	878 (21.23%)	810 (19.25%)	
High school graduate	1799 (21.56%)	959 (23.19%)	840 (19.97%)	
Has more than a high school education	4854 (58.18%)	2297 (55.54%)	2557 (60.78%)	
Poverty to income ratio	2.51 (1.66)	2.57 (1.66)	2.45 (1.65)	0.001
**Marital status, n (%)**				<0.001
Married	4200 (50.34%)	2259 (54.62%)	1941 (46.14%)	
Never married	1701 (20.39%)	893 (21.59%)	808 (19.21%)	
Others	2442 (29.27%)	984 (23.79%)	1458 (34.66%)	
**PHQ-9**	3.28 (4.55)	2.71 (4.15)	3.84 (4.85)	<0.001
Log MMA, μmol/L	5.00 (0.49)	5.01 (0.48)	4.98 (0.50)	<0.001
**BMI, kg/m2**	29.10 (7.02)	28.52 (6.12)	29.67 (7.77)	<0.001
Vitamin B12, pmol/L	463.79 (423.49)	433.84 (311.80)	493.24 (508.31)	<0.001
**Folate**, μmol/L	1178.24 (547.58)	1130.86 (500.17)	1224.81 (586.83)	<0.001
**MCV, fL**	89.38 (5.93)	89.97 (5.43)	88.80 (6.32)	<0.001
HCT	41.14 (4.19)	43.41 (3.65)	38.91 (3.41)	<0.001
**Mortality, n (%)**				0.001
Assumed deceased	708 (8.49%)	395 (9.55%)	313 (7.44%)	
Assumed alive	7635 (91.51%)	3741 (90.45%)	3894 (92.56%)	
**Follow up time, months, median (SE)**	80.00 (17.73)	80.50 (18.25)	80.00 (17.21)	0.649

*SD* standard deviation, *PHQ-9* the Patient Health Questionnaire, *MMA* methylmalonic acid, *BMI* body weight index, *MCV* mean cell volume, *HCT* Hematocrit.

### The association between methylmalonic acid and depressive symptoms

**Fig. 1:**
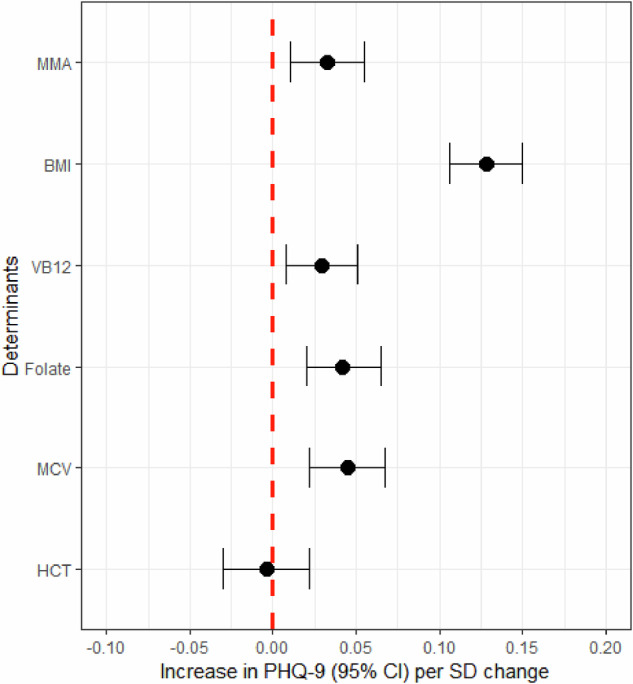
The associations of methylmalonic acid and depressive symptom. MMA methylmalonic acid, BMI body weight index, MCV mean cell volume, HCT Hematocrit. Lines represent change in PHQ-9 (beta estimate) with 95% confidence interval each standard deviation in MMA from linear regression models adjusted for age, sex, race/ethnicity, educational attainment, marital status, poverty income ratio, BMI, VB12, mean cell volume, and hematocrit. Degree of freedom = 8332.

Figure 1 and Supplementary Table 1 show that each SD (around 0.49 μmol/L) increase in MMA was associated with a 0.03 SD (approximately 0.15 score) increase in depressive symptoms (β = 0.024, 95% CI: 0.002, 0.047, *p* = 0.034) adjusting for age, sex, race/ethnicity, education, poverty income ratio, and marital status. After additional adjustment for BMI, vitamin B12, folate, MCV, and hematocrit, the association between MMA and depressive symptoms remained (β = 0.033, 95% CI: 0.010, 0.055, *p* = 0.005), which indicates that the association between MMA and depressive symptoms was independent of vitamin B12 and folate levels (Fig. 1). Moreover, sensitivity analyses indicate that the association between MMA and depressive symptoms (β = 0.026, 95% CI: 0.003 to 0.049, *p* = 0.028) is robust, holding steady even after accounting for the dietary intake of vitamin B12 and folate (Supplementary Table 2).

### Stratified analysis by sex and age

**Fig. 2:**
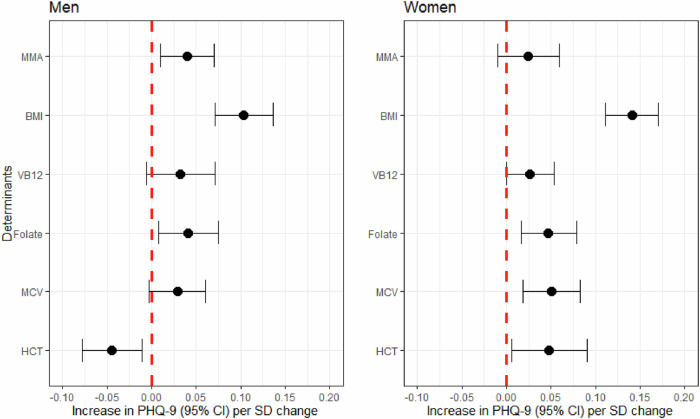
Association between methylmalonic acid and depressive symptom stratified by sex. MMA methylmalonic acid, BMI body weight index, VB12 Vitamin B12, MCV mean cell volume, HCT Hematocrit.; Lines represent change in PHQ-9 (beta estimate) with 95% confidence interval each standard deviation in MMA from linear regression models adjusted for age, race/ethnicity, educational attainment, marital status, poverty income ratio, BMI, VB12, mean cell volume, and hematocrit. Degree of freedom is 4117 for men, 4190 for women.

As for sex difference, the PHQ-9 score increased by 0.04 SD (approximately 0.17 score) for each SD (approximately 0.48 μmol/L) increase in MMA among men (β = 0.040, 95% CI: 0.010, 0.070, *p* = 0.010), whereas no significant association between MMA and depressive symptoms was observed among women (β = 0.024, 95% CI: -0.010, 0.059, *p* = 0.160) (Fig. 2 and Supplementary Table 3). Moreover, the association between MMA and depressive symptoms was independent of vitamin B12 and MCV among men.

**Fig. 3:**
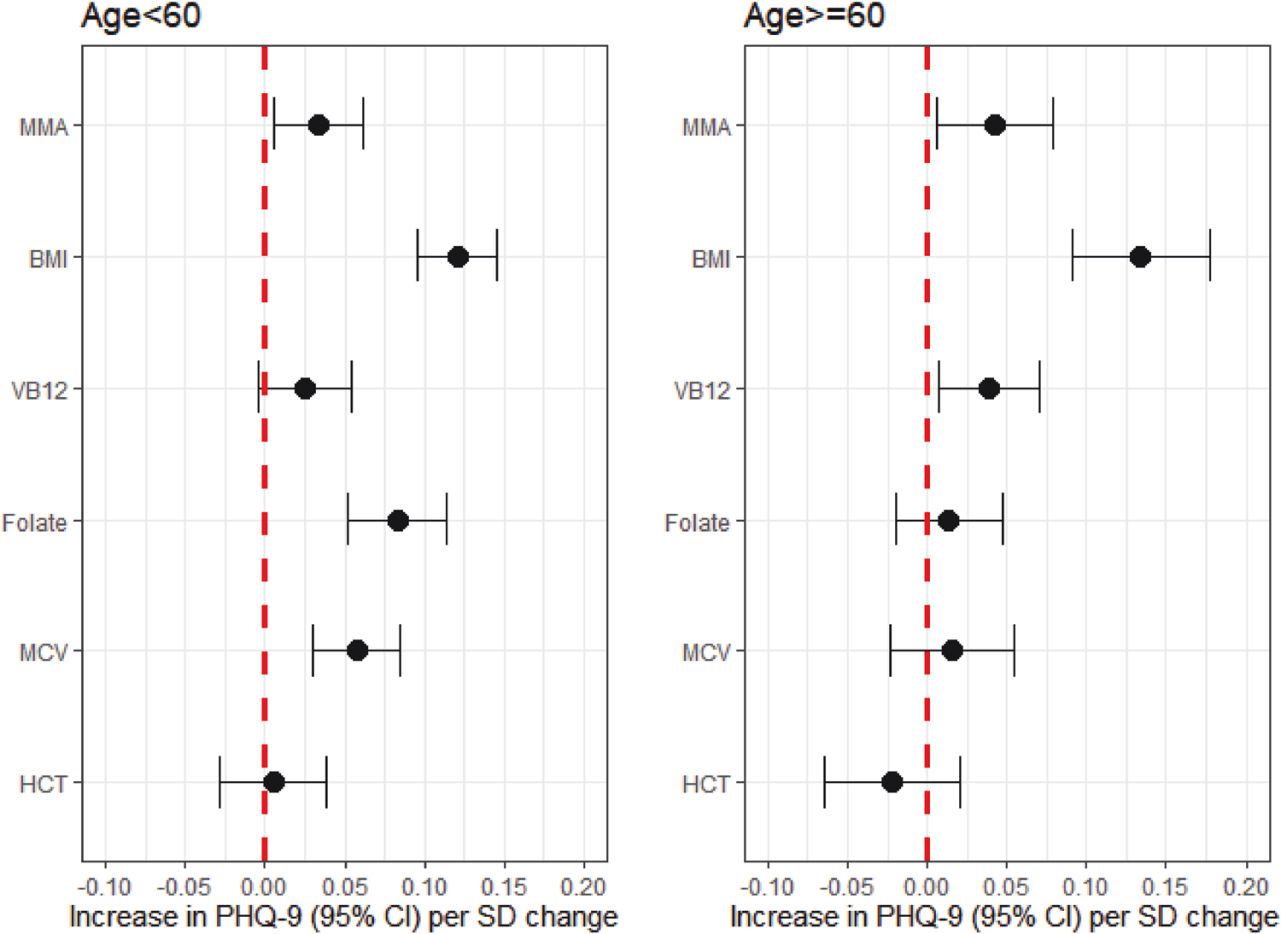
Association between methylmalonic acid and depressive symptom stratified by age. MMA methylmalonic acid, BMI body weight index, VB12 Vitamin B12, MCV mean cell volume, HCT Hematocrit.; Lines represent change in PHQ-9 (beta estimate) with 95% confidence interval each standard deviation in MMA from linear regression models adjusted for sex, race/ethnicity, educational attainment, marital status, poverty income ratio, BMI, VB12, mean cell volume, and hematocrit. Degree of freedom is 5657 for individuals with age < 60, 2650 for individuals with age >= 60.

Figure 3 and Supplementary Table 4 show that the association between MMA and depressive symptoms was more prominent in those aged older than 60 (β = 0.043, 95% CI: 0.006, 0.079, *p* = 0.021). For the elderly population, each SD (around 0.53 μmol/L) increase in MMA was significantly associated with a 0.04 SD (approximately 0.18 score) increase in depressive symptoms (β = 0.043, 95% CI: 0.006, 0.079, *p* = 0.021). Among younger participants, the association between MMA and depressive symptoms was slightly weaker (β = 0.033, 95% CI: 0.005, 0.062, *p* = 0.022).

### The association of methylmalonic acid and depressive symptoms with mortality

**Fig. 4:**
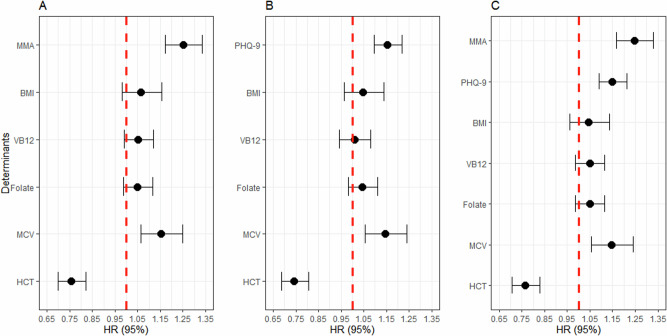
The associations of methylmalonic acid and depressive symptom with mortality. **A** Association between methylmalonic acid and mortality; **B** association between depressive symptom and mortality; **C** association between MMA and mortality controlling depressive symptom. PHQ-9 the Patient Health Questionnaire, MMA methylmalonic acid, BMI body weight index, VB12 Vitamin B12, MCV mean cell volume, HCT Hematocrit.; Lines represent change in mortality [hazard ratio (HR)] with 95% confidence interval (CI) each standard deviation change in MMA/PHQ-9 from Cox proportional hazards model adjusted for age, sex, race/ethnicity, educational attainment, marital status, poverty income ratio, BMI, VB12, mean cell volume, and hematocrit. Wald test was used. Degree of freedom = 8341.

**Fig. 5:**
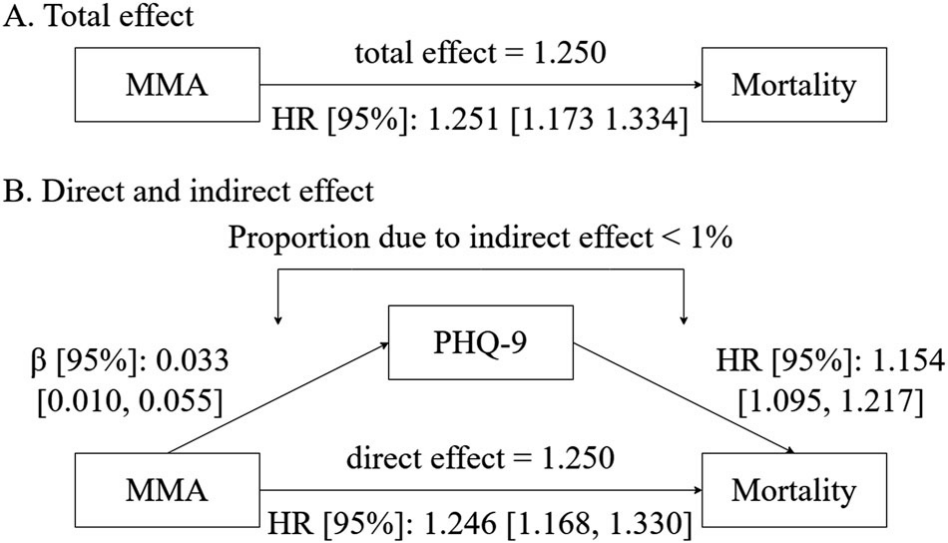
Direct and indirect effects of methylmalonic acid on mortality with depressive symptom as mediator. PHQ-9 the Patient Health Questionnaire, MMA methylmalonic acid. Models were adjusted for age, sex, race/ethnicity, educational attainment, marital status, poverty income ratio, BMI, VB12, mean cell volume, and hematocrit.

Among 8,343 participants, a total of 708 deaths were observed during a median follow-up period of 80.00 months. The effects of MMA and depressive symptoms on all-cause mortality are shown in Fig. 4. Figure 4A presents that for each SD (around 0.49 μmol/L) increase in MMA, the risk of mortality increases by 25.1% (HR = 1.251, 95% CI: 1.173, 1.334, *p* < 0.001), after adjusting for BMI, vitamin B12, folate, MCV, hematocrit, and sociodemographic factors. Similarly, Fig. 4B demonstrates that the risk of mortality exhibits an increase of 15.4% for every SD (around 4.55 scores) increase in depressive symptoms (HR = 1.154, 95% CI: 1.095, 1.217, *p* < 0.001). Moreover, MMA remained associated with a higher risk of all-cause mortality (HR = 1.246, 95% CI: 1.168, 1.330, *p* < 0.001) after accounting for the depressive symptoms. The inclusion of depressive symptoms accounted for approximately 2.0% of the association between MMA and the risk of mortality. However, no obvious mediating effect of depressive symptoms on the association between MMA and the risk of mortality was observed (Fig. 5 and Supplementary Table 6).

## Discussion

In this study of 8343 participants from NHANES 2011-2014, we found that 1) MMA was significantly associated with depressive symptoms with notable variations by sex and age groups: specifically, the association between MMA and depressive symptoms was observed only among men and it was more pronounced in individuals aged 60 and above; 2) both MMA and depressive symptoms were associated with higher risks of mortality; 3) depressive symptoms accounted for approximately 2.0% of the association between MMA and the risk of mortality, but no significant mediating effect was observed.

Our study demonstrated the association between MMA and depressive symptoms in the general population, which is in line with previous research in specific populations [12, 13]. Mitochondrial dysfunction is one of the possible mechanisms underlying the association between MMA and depression. MMA is considered a biomarker of mitochondrial dysfunction that disrupts the redox balance [28]. The “mitochondria theory of depression” contends that mitochondria, which provide energy for all cell functions, mediate the biological mechanisms underlying depression, including those associated with monoamines and neural plasticity [5, 29]. With impairing mitochondrial function, elevated levels of MMA may cause neuronal damage and contribute to depressive symptoms. The disturbance of mitochondria leads to oxidative stress, which contributes to depression through altering brain structure [6]. Previous studies have revealed that MMA induces neuron apoptosis and might lead to oxidative stress [30–32]. This suggests that the association between MMA and depression could perhaps be attributed to mitochondrial dysfunction and oxidative stress. Also, high MMA may inhibit glutamatergic neurotransmission by disrupting neuronal mitochondria [28]. In conclusion, elevated MMA may contribute to the etiology of depression through these mechanisms.

Moreover, we discovered that vitamin B12 and folate were related to depressive symptoms, in line with previous findings [33, 34]. The effect of vitamin B12 and folate deficiency on depression can be explained by one-carbon metabolism, which is crucial for methylation reactions and brain tissue and functions [19]. Generated from a key one-carbon metabolism intermediate (i.e., methylmalonyl-CoA), elevated MMA indicates dysfunction in one-carbon metabolism and can lead to detrimental effects on methylation reactions. But most importantly, our results showed that MMA was associated with depressive symptoms independent of these factors. This indicates that beyond the one-carbon metabolism, MMA-triggered mechanisms (i.e., mitochondrial, oxidative stress, and inflammation) may also contribute to depression [28].

In alignment with prior research, the present study has provided evidence that both MMA and depressive symptoms confer mortality risk. A large prospective study found a strong correlation between a baseline MMA level and an increased risk of long-term all-cause and cardiovascular mortality [17]. The effect of depression on mortality has been proven among different populations [2, 3]. However, this study failed to find a mediation result, suggesting that MMA-associated depressive symptoms are less likely to meaningfully heighten mortality risk. This could be attributed to the fact that the impact of MMA on deaths appears to be primarily influenced by physiological disturbances. Mitochondrial dysfunction can be one of the mechanisms underlying the association between MMA and chronic diseases, which may lead to death [35, 36]. Furthermore, we observed that MMA and related variables accounted for only a marginal proportion of variance (9%) in depressive symptoms. This discovery suggests that MMA-related biochemical pathways are just one of many factors that contribute to the complex, multifaceted pathophysiology of depression.

Additionally, there are sex and age differences in the association between MMA and depressive symptoms. Previous research that found a significant connection between MMA and depression was based on samples from female subgroups: a study of 700 disabled, non-demented women over 65 years old reported that those who were depressed had a significantly higher serum methylmalonic acid level (p = 0.03) [13]; additional research of Indian postpartum women found that rising MMA levels (OR = 2.14; 95%CI: 1.63-2.83) were significantly linked to a higher risk of postpartum depression [12]. Nevertheless, we failed to find a significant association between MMA and depressive symptoms in the female sample. This may be due to the use of different focus groups or measures of depressive symptoms. Yet sex differences may also exist. According to a study based on NHANES III, homocysteine levels were found to be higher in boys than in girls throughout adolescence [37]. The findings suggest that male adolescents may be more affected by one-carbon metabolic disruption than female adolescents, which may be one explanation for the sex difference.

Our study also showed that those aged 60 and older had a stronger association between MMA and depressive symptoms, suggesting that older people were more susceptible to MMA. In recent years, there has been a growing interest in MMA as a potential biomarker for aging, as its concentration is reported to increase with age beyond accepted reference ranges [28, 38, 39]. Elevated levels of MMA have been identified to potentially disrupt mitochondrial energy metabolism, leading to the generation of intracellular free radicals and subsequent initiation of cellular apoptosis [28, 40]. The increase in mitochondria-derived circulating MMA levels among seniors corresponds to vitamin B12 deficiency [41, 42] and age-associated cognitive damage [16, 43], both of which are recognized as predisposing factors for depression.

Our study has several strengths. First, to our knowledge, it is the first sizable, nationally representative observational study that has examined the relationships between MMA and depressive symptoms in U.S. adults, which may increase the applicability of the findings. Furthermore, this study investigated the potential mediating effect of depressive symptoms on the association between MMA and mortality. However, it is necessary to address the limitations of our study. First, causal conclusions cannot be drawn from a cross-sectional study design. Clarification of the mechanism linking MMA to depressive symptoms requires further investigation. Another limitation is the lack of control for homocysteine, which plays a crucial part in the association between depression and vitamin B12 and folate deficiency [19]. Additionally, the data on depressive symptoms were gathered using the self-reported PHQ-9 questionnaire, which contains measurement errors. Finally, one of the limitations of our study is the absence of direct measurements for a more extensive range of specific biomarkers that indicate mitochondrial dysfunction or oxidative stress, such as lactate [44] or fibroblast growth factor 21 (FGF21) [45]. Future studies would benefit from the inclusion of these additional biomarkers to provide a better understanding of the underlying biological mechanisms at play.

## Conclusions

In conclusion, the current population-based research found a positive association between MMA and depressive symptoms in the general population, with age and sex differences. However, while both MMA and depressive symptoms increase the risk of death, depressive symptoms are less likely to mediate the link between the two. These findings imply that MMA-associated mechanisms are a part of the complex, multi-faceted etiopathogenesis of depression, yet MMA-related depressive symptoms have no remarkable mortality-related effects.

### Supplementary information

Supplementary Materials

## Data Availability

The data that support the findings of our research are publicly accessible via the National Health and Nutrition Examination Survey (NHANES) database, which can be found at https://www.cdc.gov/nchs/nhanes/index.htm.

## References

[1] Global, regional, and national burden of 12 mental disorders in 204 countries and territories, 1990-2019. a systematic analysis for the Global Burden of Disease Study 2019. Lancet Psychiatry. 2022;9:137–50. 35026139 10.1016/S2215-0366(21)00395-3 PMC8776563

[2] Meng R, Yu C, Liu N, He M, Lv J, Guo Y, et al. Association of depression with all-cause and cardiovascular disease mortality among adults in China. JAMA Network Open. 2020;3:e1921043–e43. 32049295 10.1001/jamanetworkopen.2019.21043 PMC7212017

[3] Lee SY, Lee JP, Lee J, Park JY, Kim EY. Association between depressive symptoms and the risk of all-cause and cardiovascular mortality among US adults. Prog Neuropsychopharmacol Biol Psychiatry. 2023;125:110755. 36958666 10.1016/j.pnpbp.2023.110755

[4] Raison CL, Capuron L, Miller AH. Cytokines sing the blues: inflammation and the pathogenesis of depression. Trends Immunol. 2006;27:24–31. 16316783 10.1016/j.it.2005.11.006 PMC3392963

[5] Fries GR, Saldana VA, Finnstein J, Rein T. Molecular pathways of major depressive disorder converge on the synapse. Mol Psychiatry. 2023;28:284–97. 36203007 10.1038/s41380-022-01806-1 PMC9540059

[6] Bhatt S, Nagappa AN, Patil CR. Role of oxidative stress in depression. Drug Discov Today. 2020;25:1270–76. 32404275 10.1016/j.drudis.2020.05.001

[7] Indo HP, Davidson M, Yen HC, Suenaga S, Tomita K, Nishii T, et al. Evidence of ROS generation by mitochondria in cells with impaired electron transport chain and mitochondrial DNA damage. Mitochondrion. 2007;7:106–18. 17307400 10.1016/j.mito.2006.11.026

[8] Chandler RJ, Zerfas PM, Shanske S, Sloan J, Hoffmann V, DiMauro S, et al. Mitochondrial dysfunction in mut methylmalonic acidemia. Faseb J. 2009;23:1252–61. 19088183 10.1096/fj.08-121848 PMC2660647

[9] Pettenuzzo LF, Schuck PF, Wyse AT, Wannmacher CM, Dutra-Filho CS, Netto CA, et al. Ascorbic acid prevents water maze behavioral deficits caused by early postnatal methylmalonic acid administration in the rat. Brain Res. 2003;976:234–42. 12763258 10.1016/S0006-8993(03)02722-7

[10] Pettenuzzo LF, Ferreira Gda C, Schmidt AL, Dutra-Filho CS, Wyse AT, Wajner M. Differential inhibitory effects of methylmalonic acid on respiratory chain complex activities in rat tissues. Int J Dev Neurosci. 2006;24:45–52. 16324816 10.1016/j.ijdevneu.2005.10.005

[11] Schuck PF, Rosa RB, Pettenuzzo LF, Sitta A, Wannmacher CM, Wyse AT, et al. Inhibition of mitochondrial creatine kinase activity from rat cerebral cortex by methylmalonic acid. Neurochem Int. 2004;45:661–7. 15234108 10.1016/j.neuint.2004.03.006

[12] Dhiman P, Pillai RR, Wilson AB, Premkumar N, Bharadwaj B, Ranjan VP, et al. Cross-sectional association between vitamin B12 status and probable postpartum depression in Indian women. BMC Pregnancy Childbirth. 2021;21:146. 33596868 10.1186/s12884-021-03622-x PMC7890831

[13] Penninx BW, Guralnik JM, Ferrucci L, Fried LP, Allen RH, Stabler SP. Vitamin B(12) deficiency and depression in physically disabled older women: epidemiologic evidence from the Women’s Health and Aging Study. Am J Psychiatry. 2000;157:715–21. 10784463 10.1176/appi.ajp.157.5.715

[14] Clarke R, Birks J, Nexo E, Ueland PM, Schneede J, Scott J, et al. Low vitamin B-12 status and risk of cognitive decline in older adults. Am J Clin Nutr. 2007;86:1384–91. 17991650 10.1093/ajcn/86.5.1384

[15] Pascoe MC, Linden T. Folate and MMA predict cognitive impairment in elderly stroke survivors: a cross sectional study. Psychiatry Res. 2016;243:49–52. 27367490 10.1016/j.psychres.2016.06.008

[16] McCracken C, Hudson P, Ellis R, McCaddon A. Methylmalonic acid and cognitive function in the Medical Research Council Cognitive Function and Ageing Study. Am J Clin Nutr. 2006;84:1406–11. 17158424 10.1093/ajcn/84.6.1406

[17] Wang S, Liu Y, Liu J, Tian W, Zhang X, Cai H, et al. Mitochondria-derived methylmalonic acid, a surrogate biomarker of mitochondrial dysfunction and oxidative stress, predicts all-cause and cardiovascular mortality in the general population. Redox Biol. 2020;37:101741. 33035815 10.1016/j.redox.2020.101741 PMC7554255

[18] Products EPoD. Scientific opinion on dietary reference values for cobalamin (vitamin B12). EFSA J. 2015;13:16–22. 10.2903/j.efsa.2015.4150 PMC13151598 42109795

[19] Frankenburg FR. The role of one-carbon metabolism in schizophrenia and depression. Harv Rev Psychiatry. 2007;15:146–60. 17687709 10.1080/10673220701551136

[20] Folstein M, Liu T, Peter I, Buell J, Arsenault L, Scott T, et al. The homocysteine hypothesis of depression. Am J Psychiatry. 2007;164:861–7. 17541043 10.1176/ajp.2007.164.6.861

[21] Sachdev PS, Parslow RA, Lux O, Salonikas C, Wen W, Naidoo D, et al. Relationship of homocysteine, folic acid and vitamin B12 with depression in a middle-aged community sample. Psychol Med. 2005;35:529–38. 15856723 10.1017/S0033291704003721

[22] Esnafoglu E, Ozturan DD. The relationship of severity of depression with homocysteine, folate, vitamin B12, and vitamin D levels in children and adolescents. Child Adolesc Ment Health. 2020;25:249–55. 32304285 10.1111/camh.12387

[23] Kroenke K, Spitzer RL, Williams JB. The PHQ‐9: validity of a brief depression severity measure. J General Internal Med. 2001;16:606–13. 10.1046/j.1525-1497.2001.016009606.x PMC1495268 11556941

[24] Hamer M, Bates CJ, Mishra GD. Depression, physical function, and risk of mortality: National Diet and Nutrition Survey in adults older than 65 years. Am J Geriatr Psychiatry. 2011;19:72–8. 20808095 10.1097/JGP.0b013e3181df465e

[25] Hamer M, Molloy GJ, Stamatakis E. Psychological Distress as a Risk Factor for Cardiovascular Events: Pathophysiological and Behavioral Mechanisms. J Am College Cardiol. 2008;52:2156–62. 10.1016/j.jacc.2008.08.057 19095133

[26] Valeri L, VanderWeele TJ. Mediation analysis allowing for exposure–mediator interactions and causal interpretation: Theoretical assumptions and implementation with SAS and SPSS macros. Psycholog Methods. 2013;18:137–50. 10.1037/a0031034 PMC3659198 23379553

[27] Valeri L, VanderWeele TJ. SAS macro for causal mediation analysis with survival data. Epidemiology. 2015;26:e23–e24. 25643116 10.1097/EDE.0000000000000253

[28] Liu Y, Wang S, Zhang X, Cai H, Liu J, Fang S, et al. The regulation and characterization of mitochondrial-derived methylmalonic acid in mitochondrial dysfunction and oxidative stress: from basic research to clinical practice. Oxidative Med Cellular Longevity. 2022;2022:7043883. 10.1155/2022/7043883 PMC9155905 35656023

[29] Hasin DS, Sarvet AL, Meyers JL, Saha TD, Ruan WJ, Stohl M, et al. Epidemiology of adult DSM-5 major depressive disorder and its specifiers in the United States. JAMA Psychiatry. 2018;75:336–46. 29450462 10.1001/jamapsychiatry.2017.4602 PMC5875313

[30] Fernandes CG, Borges CG, Seminotti B, Amaral AU, Knebel LA, Eichler P, et al. Experimental evidence that methylmalonic acid provokes oxidative damage and compromises antioxidant defenses in nerve terminal and striatum of young rats. Cell Mol Neurobiol. 2011;31:775–85. 21424830 10.1007/s10571-011-9675-4 PMC11498429

[31] Richard E, Alvarez-Barrientos A, Pérez B, Desviat LR, Ugarte M. Methylmalonic acidaemia leads to increased production of reactive oxygen species and induction of apoptosis through the mitochondrial/caspase pathway. J Pathol. 2007;213:453–61. 17948227 10.1002/path.2248

[32] Proctor EC, Turton N, Boan EJ, Bennett E, Philips S, Heaton RA, et al. The effect of methylmalonic acid treatment on human neuronal cell coenzyme Q(10) status and mitochondrial function. Int J Mol Sci. 2020;21:9137. 33266298 10.3390/ijms21239137 PMC7730949

[33] Petridou ET, Kousoulis AA, Michelakos T, Papathoma P, Dessypris N, Papadopoulos FC, et al. Folate and B12 serum levels in association with depression in the aged: a systematic review and meta-analysis. Aging Ment Health. 2016;20:965–73. 26055921 10.1080/13607863.2015.1049115

[34] Kaplan BJ, Crawford SG, Field CJ, Simpson JS. Vitamins, minerals, and mood. Psychol Bull. 2007;133:747–60. 17723028 10.1037/0033-2909.133.5.747

[35] Mc Guire PJ, Parikh A, Diaz GA. Profiling of oxidative stress in patients with inborn errors of metabolism. Mol Genet Metab. 2009;98:173–80. 19604711 10.1016/j.ymgme.2009.06.007 PMC2915835

[36] Wang J, Tang Y, Liu Y, Cai W, Xu J. Correlations between circulating methylmalonic acid levels and all-cause and cause-specific mortality among patients with diabetes. Front Nutr. 2022;9:974938. 36523337 10.3389/fnut.2022.974938 PMC9745031

[37] Must A, Jacques PF, Rogers G, Rosenberg IH, Selhub J. Serum total homocysteine concentrations in children and adolescents: results from the third National Health and Nutrition Examination Survey (NHANES III). J Nutr. 2003;133:2643–9. 12888652 10.1093/jn/133.8.2643

[38] Lewerin C, Ljungman S, Nilsson-Ehle H. Glomerular filtration rate as measured by serum cystatin C is an important determinant of plasma homocysteine and serum methylmalonic acid in the elderly. J Intern Med. 2007;261:65–73. 17222169 10.1111/j.1365-2796.2006.01732.x

[39] Gomes AP, Ilter D, Low V, Endress JE, Fernández-García J, Rosenzweig A, et al. Age-induced accumulation of methylmalonic acid promotes tumour progression. Nature. 2020;585:283–87. 32814897 10.1038/s41586-020-2630-0 PMC7785256

[40] Luciani A, Devuyst O. Methylmalonyl acidemia: from mitochondrial metabolism to defective mitophagy and disease. Autophagy. 2020;16:1159–61. 32316822 10.1080/15548627.2020.1753927 PMC7469617

[41] Bailey RL, Carmel R, Green R, Pfeiffer CM, Cogswell ME, Osterloh JD, et al. Monitoring of vitamin B-12 nutritional status in the United States by using plasma methylmalonic acid and serum vitamin B-12. Am J Clin Nutr. 2011;94:552–61. 21677051 10.3945/ajcn.111.015222 PMC3142730

[42] Jarquin Campos A, Risch L, Nydegger U, Wiesner J, Vazquez Van Dyck M, Renz H, et al. Diagnostic accuracy of holotranscobalamin, vitamin B12, methylmalonic acid, and homocysteine in detecting B12 deficiency in a large, mixed patient population. Dis Markers. 2020;2020:7468506. 32089757 10.1155/2020/7468506 PMC7017578

[43] Vogiatzoglou A, Smith AD, Nurk E, Drevon CA, Ueland PM, Vollset SE, et al. Cognitive function in an elderly population: interaction between vitamin B12 status, depression, and apolipoprotein E ε4: the Hordaland Homocysteine Study. Psychosom Med. 2013;75:20–9. 23213264 10.1097/PSY.0b013e3182761b6c

[44] Jeanson Y, Ribas F, Galinier A, Arnaud E, Ducos M, André M, et al. Lactate induces FGF21 expression in adipocytes through a p38-MAPK pathway. Biochem J. 2016;473:685–92. 26769382 10.1042/BJ20150808

[45] Forsström S, Jackson CB, Carroll CJ, Kuronen M, Pirinen E, Pradhan S, et al. Fibroblast growth factor 21 drives dynamics of local and systemic stress responses in mitochondrial myopathy with mtDNA Deletions. Cell Metab. 2019;30:1040–54.e7. 31523008 10.1016/j.cmet.2019.08.019

