# Supplementary material for: Associations of methylmalonic acid and depressive symptoms with mortality: a population-based study
Source: Transl Psychiatry. 2024 Jul 19;14:297. doi: 10.1038/s41398-024-03015-6 (PMC11271623; doi:10.1038/s41398-024-03015-6)
Supplement: Supplementary file 1 — Supplementary Materials [file 41398_2024_3015_MOESM1_ESM.docx]

**Supplementary Materials**

**Table S1. Multivariable linear regression analysis examining the association between methylmalonic acid and depressive symptoms (PHQ-9).**

**Table S2. Multivariable linear regression analysis examining the association between methylmalonic acid and depressive symptoms adjusted for dietary intake.**

**Table S3. Multivariable linear regression analysis examining the association between methylmalonic acid and depressive symptoms stratified by sex.**

**Table S4. Multivariable linear regression analysis examining the association between methylmalonic acid and depressive symptoms stratified by age.**

**Table S5. Multivariable Cox proportional hazard regression examining the association between methylmalonic acid, depressive symptoms (PHQ-9) and mortality.**

**Table S6. Direct and indirect effects of methylmalonic acid on mortality with depressive symptom as mediator.Figure S1. Flow chart of the participant selection.**

**Figure S1. Flow chart of participant selection.**

**Figure S2. Correlation matrix of methylmalonic acid and depressive symptoms.**

**Table S1. Multivariable linear regression analysis examining the association between methylmalonic acid and depressive symptom.**

|  | Estimate | 95% CI | P-value | R^2^ |
| --- | --- | --- | --- | --- |
| Model1 | 0.024 | (0.002, 0.047) | 0.034 | 0.072 |
| Model2 | 0.025 | (0.002, 0.047) | 0.030 | 0.086 |
| Model3 | 0.033 | (0.010, 0.056) | 0.005 | 0.088 |
| Model4 | 0.033 | (0.010, 0.055) | 0.005 | 0.090 |
| Abbreviations: PHQ-9, the Patient Health Questionnaire; MMA, methylmalonic acid; BMI, body weight index; VB12, Vitamin B12; MCV, mean cell volume; HCT, Hematocrit.;  Model 1: adjusted for sex, race/ethnicity, education, poverty-to-income ratio, and marital status;  Model 2: model 1 + BMI;  Model 3: model 2 + Vitamin B12 + Folate;  Model 4: model 3 + MCV + HCT. | | | | |

**Table S2. Multivariable linear regression analysis examining the association between methylmalonic acid and depressive symptoms adjusted for dietary intake.**

|  | Estimate | 95%CI | P-value |  |
| --- | --- | --- | --- | --- |
| MMA | 0.026 | (0.003,0.049) | 0.028 |  |
| BMI | 0.128 | (0.106,0.150) | <0.001 |  |
| Dietary VB12 | 0.000 | (-0.023,0.022) | 0.993 |  |
| Dietary Folate | -0.017 | (-0.039,0.006) | 0.153 |  |
| MCV | 0.046 | (0.023,0.068) | <0.001 |  |
| HCT | 0.001 | (-0.025,0.027) | 0.944 |  |
| Abbreviations: PHQ-9, the Patient Health Questionnaire; MMA, methylmalonic acid; BMI, body weight index; VB12, Vitamin B12; MCV, mean cell volume; HCT, Hematocrit.; Model was adjusted for sex, race/ethnicity, education, poverty-to-income ratio, and marital status. | | | |  |
|  |  |  |  |  |

**Table S3. Multivariable linear regression analysis examining the association between methylmalonic acid and depressive symptom stratified by sex.**

|  | Men (N = 2495) | | | | Women (N = 3031) | | | |
| --- | --- | --- | --- | --- | --- | --- | --- | --- |
|  | Estimate | 95%CI | P-value | R^2^ | Estimate | 95%CI | P-value | R^2^ |
| Model1 | 0.034 | (0.004, 0.063) | 0.026 | 0.051 | 0.016 | (-0.018, 0.049) | 0.370 | 0.068 |
| Model2 | 0.035 | (0.005, 0.064) | 0.022 | 0.060 | 0.015 | (-0.018, 0.049) | 0.371 | 0.086 |
| Model3 | 0.043 | (0.012, 0.073) | 0.006 | 0.063 | 0.024 | (-0.010, 0.058) | 0.169 | 0.089 |
| Model4 | 0.040 | (0.010, 0.070) | 0.010 | 0.065 | 0.024 | (-0.010, 0.059) | 0.160 | 0.093 |
| Abbreviations: PHQ-9, the Patient Health Questionnaire; MMA, methylmalonic acid; BMI, body weight index; VB12, Vitamin B12; MCV, mean cell volume; HCT, Hematocrit.;  Model 1: adjusted for age, race/ethnicity, education, poverty-to-income ratio, and marital status;  Model 2: model 1 + BMI;  Model 3: model 2 + Vitamin B12 + Folate;  Model 4: model 3 + MCV + HCT. | | | | | | | | |

**Table S4. Multivariable linear regression analysis examining the association between methylmalonic acid and depressive symptom stratified by age.**

|  | Age < 60 years (N = 5674) | | | | Age >= 60 years (N = 2669) | | | |
| --- | --- | --- | --- | --- | --- | --- | --- | --- |
|  | Estimate | 95%CI | P-value | R^2^ | Estimate | 95%CI | P-value | R^2^ |
| Model1 | 0.030 | (0.002, 0.059) | 0.038 | 0.085 | 0.037 | (0.002, 0.073) | 0.039 | 0.066 |
| Model2 | 0.029 | (0.001, 0.057) | 0.044 | 0.100 | 0.036 | (0.001, 0.071) | 0.046 | 0.078 |
| Model3 | 0.036 | (0.007, 0.064) | 0.014 | 0.105 | 0.045 | (0.009, 0.081) | 0.014 | 0.081 |
| Model4 | 0.033 | (0.005, 0.062) | 0.022 | 0.108 | 0.043 | (0.006, 0.079) | 0.021 | 0.082 |
| Abbreviations: PHQ-9, the Patient Health Questionnaire; MMA, methylmalonic acid; BMI, body weight index; VB12, Vitamin B12; MCV, mean cell volume; HCT, Hematocrit.;  Model 1: adjusted for sex, race/ethnicity, education, poverty-to-income ratio, and marital status;  Model 2: model 1 + BMI;  Model 3: model 2 + Vitamin B12 + Folate;  Model 4: model 3 + MCV + HCT. | | | | | | | | |

**Table S5. Multivariable Cox proportional hazard regression examining the association between methylmalonic acid, depressive symptoms (PHQ-9) and mortality.**

|  |  | Model 1 |  |  | Model 2 |  |  | Model 3 |  |  |
| --- | --- | --- | --- | --- | --- | --- | --- | --- | --- | --- |
|  | HR | 95%CI | P-value | HR | 95%CI | P-value | HR | 95%CI | P-value |  |
| MMA | 1.237 | (1.159,1.321) | <0.001 |  | | | 1.235 | (1.156,1.320) | <0.001 |  |
| PHQ-9 |  | | | 1.162 | (1.102,1.225) | <0.001 | 1.157 | (1.097,1.220) | <0.001 |  |
| BMI | 1.086 | (1.000,1.180) | 0.051 | 1.071 | (0.984,1.165) | 0.112 | 1.065 | (0.979,1.158) | 0.142 |  |
| Dietary VB12 | 1.000 | (0.932,1.073) | 0.998 | 0.998 | (0.927,1.074) | 0.952 | 1.000 | (0.934,1.071) | 0.996 |  |
| Dietary Folate | 0.907 | (0.825,0.997) | 0.044 | 0.908 | (0.826,0.998) | 0.046 | 0.912 | (0.829,1.002) | 0.055 |  |
| MCV | 1.166 | (1.073,1.266) | <0.001 | 1.147 | (1.056,1.246) | 0.001 | 1.155 | (1.063,1.255) | <0.001 |  |
| HCT | 0.747 | (0.688,0.812) | <0.001 | 0.737 | (0.679,0.800) | <0.001 | 0.752 | (0.693,0.816) | <0.001 |  |
| Abbreviations: HR, hazard ratio; PHQ-9, the Patient Health Questionnaire; MMA, methylmalonic acid; BMI, body weight index; VB12, Vitamin B12; MCV, mean cell volume; HCT, Hematocrit.; Models were adjusted for sex, race/ethnicity, education, poverty-to-income ratio, and marital status. | | | | | | | | | |  |
|  |  |  |  |  |  |  |  |  |  |  |

**Table S6. Direct and indirect effects of** **methylmalonic acid on mortality with depressive symptom as mediator.**

|  | Estimate | 95%CI | P-value |
| --- | --- | --- | --- |
| Total effect | 1.25 | (1.17, 1.34) |  |
| Direct effect | 1.25 | (1.17, 1.33) |  |
| Proportion of indirect effect (%) | < 1.00 |  |  |
| Models were adjusted for age, sex, race/ethnicity, educational attainment, marital status, poverty income ratio, BMI, VB12, mean cell volume, and hematocrit. | | | |

**
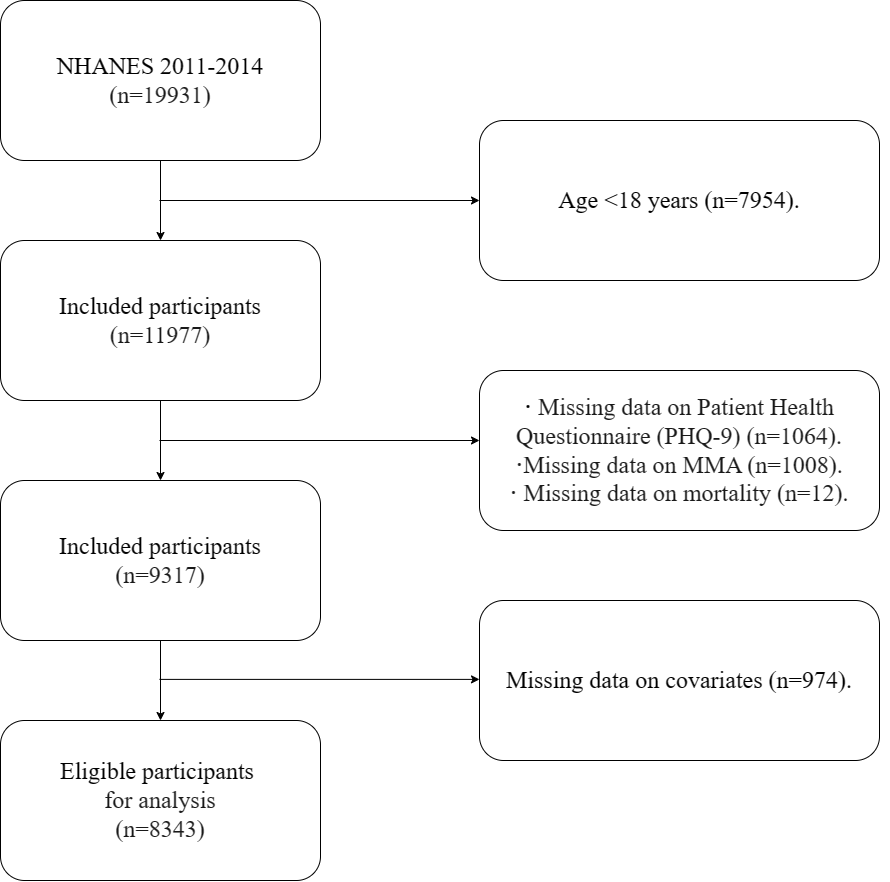
**

**Figure S1. Flow chart of participant selection.**


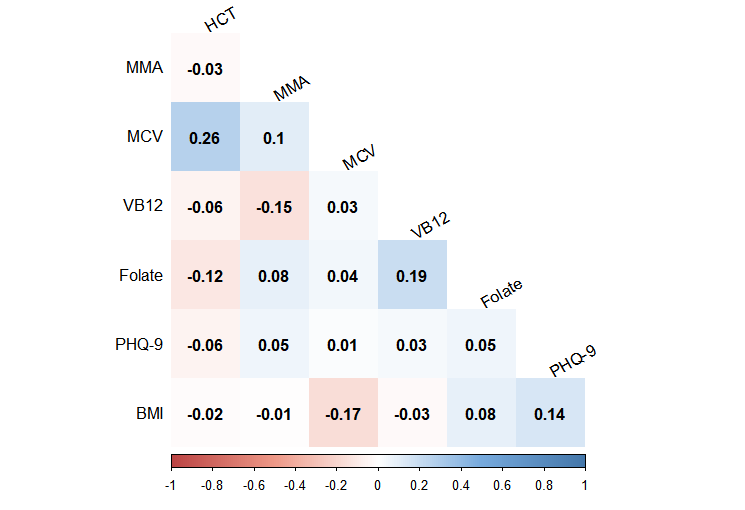


**Figure S2. Correlation matrix of methylmalonic acid, and depressive symptom.**

Abbreviations: PHQ-9, the Patient Health Questionnaire; MMA, methylmalonic acid; BMI, body weight index; VB12, Vitamin B12; MCV, mean cell volume; HCT, Hematocrit.;

Color and number present the Pearson correlation coefficients. None of the correlations of these variables were significant.
